# Reduced antibody response to COVID-19 vaccine composed of inactivated SARS-CoV-2 in diabetic individuals

**DOI:** 10.3389/fpubh.2022.1025901

**Published:** 2022-12-08

**Authors:** Yandong Cheng, Ping Shen, Yue Tao, Wenjun Zhang, Biyun Xu, Yan Bi, Zhen Han, Yi-Hua Zhou

**Affiliations:** ^1^Department of Endocrinology, Nanjing Drum Tower Hospital, Nanjing University Medical School, Nanjing, Jiangsu, China; ^2^Department of Laboratory Medicine, Nanjing Drum Tower Hospital, Nanjing University Medical School, Nanjing, Jiangsu, China; ^3^Department of Biomedicine Statistics, Nanjing Drum Tower Hospital, Nanjing University Medical School, Nanjing, Jiangsu, China; ^4^Department of Prevention and Health Care, Nanjing Drum Tower Hospital, Nanjing University Medical School, Nanjing, Jiangsu, China; ^5^Department of Infectious Diseases and Jiangsu Key Laboratory for Molecular Medicine, Nanjing Drum Tower Hospital, Nanjing University Medical School, Nanjing, Jiangsu, China

**Keywords:** T2DM patients, COVID-19, vaccination, inactivated SARS-CoV-2, impaired antibody response

## Abstract

**Background:**

Patients with type 2 diabetes mellitus (T2DM) are at increased risk for COVID-19 related morbidity and mortality. Antibody response to COVID-19 vaccine in T2DM patients is not very clear. The present work aims to evaluate the antibody response to the inactivated SARS-CoV-2 vaccine in this population.

**Methods:**

Two groups of subjects with no history of SARS-CoV-2 infection were included: 63 T2DM patients and 56 non-T2DM controls. Each participant received two doses of inactivated COVID-19 vaccine. IgG antibodies against the nucleocapsid (N) and spike (S) proteins of SARS-CoV-2 (anti-N/S IgG) and receptor binding domain (RBD) proteins (anti-RBD IgG) were quantitatively evaluated by the electrochemiluminescence immunoassays, respectively.

**Results:**

It was observed that the positive rates and titers of anti-N/S IgG and anti-RBD IgG in T2DM patients were significantly lower than those in controls, respectively (anti-N/S: 85.7 vs. 98.2%, *P* = 0.034; 25.48 vs. 33.58 AU/ml *P* = 0.011; anti-RBD: 85.7 vs. 96.4%, *P* = 0.044; 15.45 vs. 22.25 AU/ml, *P* = 0.019). Compared to non-T2DM subjects, T2DM patients with uncontrolled glycemia showed lower positive antibody rates and titers (anti-N/S IgG: 75% and 13.30 AU/ml; anti-RBD IgG: 75% and 11.91 AU/ml, respectively, all *P* < 0.05), while T2DM patients with controlled glycemia had similar positive antibody rates and titers (anti-N/S IgG: 94.3% and 33.65 AU/ml; and anti-RBD IgG: 94.3% and 19.82 AU/ml, respectively, all *P* > 0.05).

**Conclusion:**

In the analysis performed, the data indicate that T2DM patients with uncontrolled glycemia showed a lower level of IgG antibodies compared to non-diabetic controls and individuals with controlled glycemia when immunized with the inactivated COVID-19 vaccine.

## Introduction

Coronavirus disease 2019 (COVID-19), caused by severe acute respiratory syndrome coronavirus 2 (SARS-CoV-2), is a global healthcare crisis, since as of 30 October 2022, at least 627 million confirmed cases and 6.5 million deaths were reported globally (1). COVID-19 also affects other patients or causes other medical issues (2–7). Compared to healthy individuals, those who had underlying chronic diseases, including hypertension, type 2 diabetes mellitus (T2DM), chronic obstructive pulmonary disease, cerebrovascular or cardiovascular disease, and others have increased fatality rate after infection with COVID-19 (8–10). COVID-19 occurred in diabetic patients is usually more severe than in non-diabetic patients (11–13). Thus, diabetic patients are among the critical subpopulations for prevention of COVID-19 (14, 15).

Diabetic patients are at increased risk for various infections (16), suggesting that the immunity in diabetic patients is to some extents compromised. Studies showed that the antibody response to hepatitis B vaccine is impaired in diabetic patients (17–19). However, the antibody response to influenza vaccines appears to be not impaired in people with T2DM (20, 21). These studies indicate that diabetic patients may present different immune response to different vaccines.

Since December 2020, several COVID-19 vaccines, composed of inactivated SARS-CoV-2, mRNA encoding the full-length spike (S) protein of SARS-CoV-2, viral-vector based vaccine encoding the S protein, or recombinant S proteins, have been applied in human to prevent the pandemic of COVID-19 (22–25). Recently, several studies reported the antibody response to mRNA or viral-vector vaccine against COVID-19 in diabetic patients with inconsistent results (26–29). COVID-19 vaccines composed of inactivated SARS-CoV-2 have been demonstrated to be effective and are also widely used in the world (24, 25, 29–32). However, the immunogenicity of inactivated COVID-19 vaccine in diabetic patients is not very clear (33). The present study aims to evaluate the antibody response to the inactivated SARS-CoV-2 vaccine in this population.

## Materials and methods

### Participants

China issued the first license for COVID-19 vaccine (Aikewei, Beijing Institute of Biological Products/Sinopharm, Beijing, China) composed of inactivated SARS-CoV-2 on December 30, 2020, and the second inactivated COVID-19 vaccine (CoronaVac, Sinovac Life Sciences, Beijing, China) on February 5, 2021, for emergency use in adult individuals at the age 18–60 years. The recommended vaccination requires two vaccine doses at an interval 2–4 weeks. During the first three-month period of vaccination campaign, the vaccines were mainly used in individuals who were at the frontier lines for controlling the pandemic of COVID-19, such as healthcare worker and other populations at high risk for infection of SARS-CoV-2 (24, 25, 34). Since April 1, 2021, COVID-19 vaccines have been administered among all general populations at the age of 18–60 years, and the vaccines have been then applied in adults over 60 years old and children at the age of 3–17 years. The COVID-19 vaccines initially used in China were mainly composed of inactivated SARS-CoV-2 adsorbed on adsorbed on aluminum hydroxide adjuvant (Aikewei or CoronaVac) (35, 36).

This was a cross-sectional study. Two groups of participants with no history of SARS-CoV-2 infection were included, and each participant received two doses of inactivated COVID-19 vaccines (Aikewei or CoronaVac). The patient group was composed of the individuals with T2DM who were out-patients in the department of endocrinology at Nanjing Drum Tower Hospitals between March 10 and September 24, 2021. The diagnosis of T2DM was based on the criteria (37). The inclusion criteria included: (1) ≥18 years older, (2) immunized with two doses of COVID-19 vaccine composed of inactivated SARS-CoV-2, within 2–10 weeks before recruitment. Patients who met any of following conditions were excluded from the study: (1) with autoimmune disease, (2) with malignant tumor, (3) with history of administration steroid hormones or other immunosuppressive agents within recent 3 months, (4) ongoing medication with any immunosuppressive agent, (5) Type 1 Diabetes, and (6) pregnancy. The control group consisted of age and sex matched subjects who had no history of diabetes and had normal fasting blood glucose; they were healthcare workers in Nanjing Drum Tower Hospital. All the subjects in the control group underwent regular yearly health examinations at least in the last 3 years, and no one showed the fasting blood glucose over 6.4 mMol/L. The inclusion and exclusion criteria were same as those mentioned above. The blood samples were collected between March 10 and September 16, 2021.

This study was approved by the institutional review board (IRB) of Nanjing Drum Tower Hospital (No. 2021-606-02). Written informed consent was obtained from each participant.

### Sample size calculation

Considering that the positive rate of anti-RBD IgG was 97% in the non-T2DM subjects based on the results of clinical trials (33, 34) and assumed 80% in the T2DM patients, we calculated that 46 patients per group would be required, with a power of 80% and a type I error rate of 0.05, by using a χ^2^-test. On the basis of an expected dropout of 10%, we planned to enroll 52 subjects per group.

### Blood sample collection

Fasting blood samples were taken by venipuncture from each participant. In addition to the necessary laboratory tests such as clinical biochemistry and glycosylated hemoglobin, serum or plasma samples left over after clinical testing were aliquoted and stored at −30°C.

### Detection of anti-SARS-CoV-2 antibody

Two chemo-luminescence immunoassay kits for anti-SARS-CoV-2 antibody, SARS-CoV-2 IgG kit and surrogate neutralization assay kit (iFlash 3000 chemiluminescence immunoassay analyzer, Shenzhen YHLO Biotech, China), were used to measure the levels of anti-SARS-CoV-2 antibodies as described elsewhere (38, 39). The SARS-CoV-2 IgG kit detects total IgG antibodies to the combination of nucleocapsid (N) and S proteins of SARS-CoV-2 (anti-N/S IgG), and the surrogate neutralization assay kit measures the IgG antibody specific to the receptor binding domain (RBD) of the S protein (anti-RBD IgG). The surrogate neutralization activity correlates well with the inhibition of SARS-CoV-2 infection in the cell culture (39). Based on the manufacturer's instructions, the measured results with values ≥10.0 arbitrary units (AU)/ml were considered positive for the antibodies, and the results below 10.0 AU/mL as negative.

### Statistical analysis

Categorical data were presented as percentages and continuous data were presented as means ± standard deviation or median (25–75th percentile). The characteristics of participants with and without diabetes were compared by unpaired Student's *t*′-test for ages, by Mann-Whitney *U*-test for time interval (days) after the 2nd vaccine dose, and by χ^2^-test for sex and the positive rates of anti-N/S IgG and anti-RBD IgG. Seropositivity and Clopper-Pearson 95% confidence intervals (CI) were calculated. The antibody levels were compared by Mann-Whitney *U*-test. The χ^2^-test was used to compare the seropositivity of anti-N/S IgG and anti-RBD IgG between the subjects without diabetes and diabetic patients with high glycemia or with controlled glycemia. The amount of anti-N/S IgG and anti-RBD IgG in the sera of vaccinated individuals with high glycemia was compared to that in vaccinated individuals without high glycemia by Kruskal-Wallis test. A two-sided *P*-value of < 0.05 was considered significant. All statistical analyses were conducted using the SPSS 25.0 (version 25.0, SPSS, Chicago, IL, USA).

## Results

### Participant characteristics

A total of 119 participants who did not have history of SARS-CoV-2 infection were included in this study. Sixty-three subjects who had been diagnosed with T2DM were divided into the patient group and 56 subjects who did not have history of T2DM were divided into the control group. The demographic characteristics and relevant variables of these two groups are presented in Table 1. Overall, there was no statistical significance in these parameters between these two groups.

**Table 1:** Comparison of demographic characteristics and antibody response between T2DM and non-T2DM participants.

**Item**	**Total, *n* = 119**	**T2DM, *n* = 63 (%)**	**Non-T2DM, *n* = 56 (%)**	**Statistics**	***P***
Sex				χ ^2^ = 2.358	0.125
Male	62 (52.1)	37 (58.7)	25 (44.6)		
Female	57 (47.9)	26 (41.3)	31 (55.4)		
Age (years)	51.0 ± 9.7	50.4 ± 11.4	51.6 ± 7.3	*t*′= 0.727	0.469
Interval after 2nd dose (days) Median (P_25_-P_75_)	32 (26, 47)	35 (26, 51)	29 (26, 40)	*Z* = −1.437	0.151
Anti-N/S IgG^*^				χ ^2^ = 4.504	0.034
≥10 AU/ml	109 (91.6)	54 (85.7)	55 (98.2)		
<10 AU/ml	10 (8.4)	9 (14.3)	1 (1.8)		
Anti-RBD IgG^*^				χ ^2^ = 4.057	0.044
≥10 AU/ml	108 (90.8)	54 (85.7)	54 (96.4)		
<10 AU/ml	11 (9.2)	9 (14.3)	2 (3.6)		

* Results with ≥10.0 AU/mL and <10.0 AU/mL indicate IgG antibody positive and negative, respectively.

N and S, nucleocapsid (N) and spike (S) proteins of SARS-CoV-2, respectively. RBD, receptor binding domain.

### Antibody response to inactivated COVID-19 vaccine in subjects with or without T2DM

Table 1 presents the results of anti-N/S IgG and anti-RBD IgG in the T2DM patients and controls. The positive rate of anti-N/S IgG in the T2DM patients was 85.7% (54/63) (95% CI 74.6, 93.3%), and the positive rate of anti-RBD IgG was also 85.7% (54/63) (95% CI 74.6, 93.3%); these 54 patients were positive for both anti-N/S IgG and anti-RBD IgG. In the controls, 98.2% (55/56) (95% CI 90.4, 100.0%) were positive for anti-N/S IgG and 96.4% (54/56) (95% CI 87.7, 100.0%) were positive for anti-RBD IgG. The positive rates of anti-N/S IgG and anti-RBD IgG in T2DM patients were significantly lower than those in the non-T2DM subjects, respectively (both *P* < 0.05) (Table 1).

**Figure 1:**
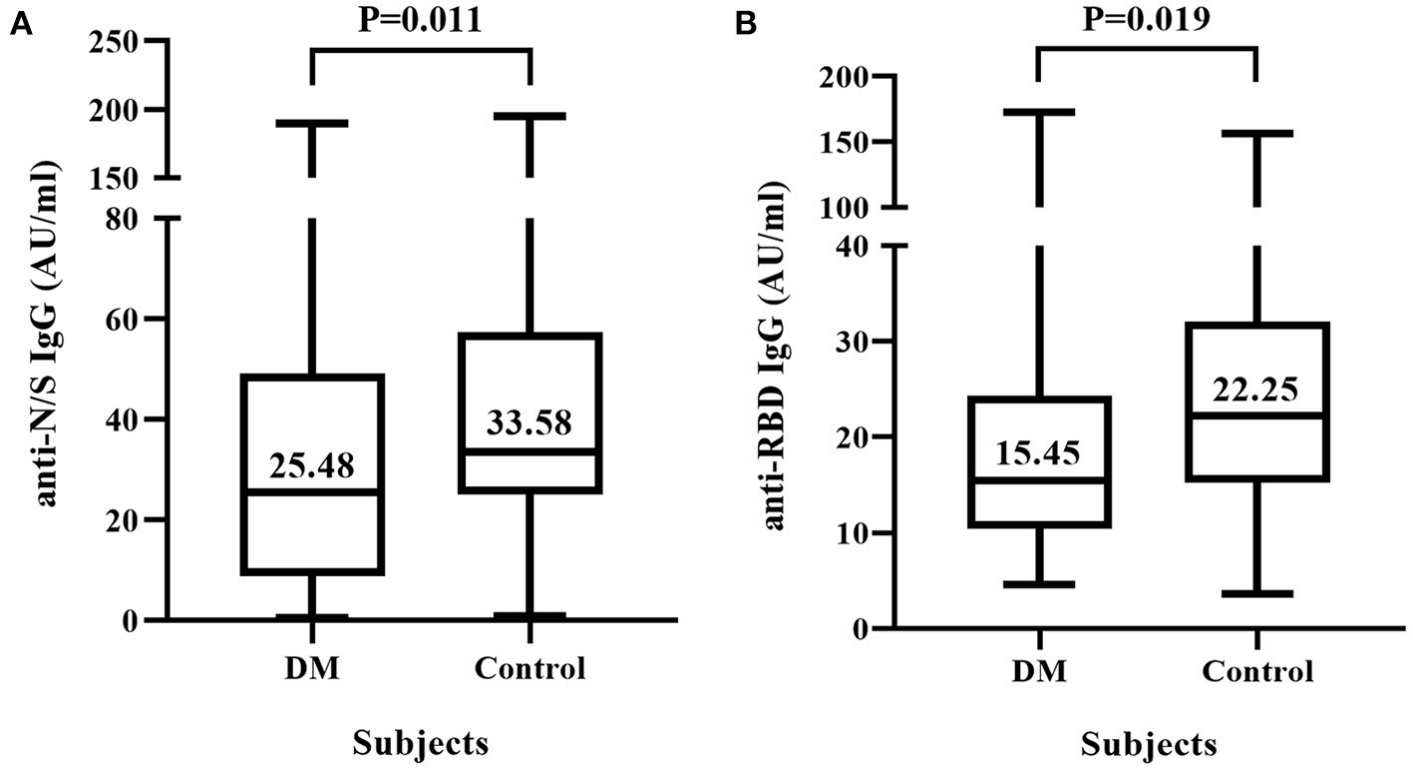
IgG antibody response to inactivated COVID-19 vaccine in diabetic mellitus (DM) patients and non-DM controls. Sixty-three DM patients and 56 non-DM subjects were each vaccinated with two doses of COVID-19 vaccine composed of inactivated SARS-CoV-2. **(A)** Titers of IgG antibody against the nucleocapsid (N) and spike (S) proteins of SARS-CoV-2 (anti-N/S IgG). **(B)** Titers of IgG antibody against receptor binding domain (RBD) of S protein (anti-RBD IgG).

As shown in Figure 1, the median (interquartile range) level of anti-N/S IgG in T2DM patients was significantly lower than that in non-T2DM subjects (25.48 [8.89, 49.14] vs. 33.58 [25.11, 57.39] AU/ml, *p* = 0.011) (Figure 1A), and similarly, the median level of anti-RBD IgG in T2DM patients was also significantly lower than that in non-T2DM subjects (15.45 [10.44, 24.34] vs. 22.25 [15.25, 32.09] AU/ml, *p* = 0.019) (Figure 1B).

### Anti-N/S IgG and anti-RBD IgG antibodies in T2DM patients with controlled and uncontrolled glycemia

**Figure 2:**
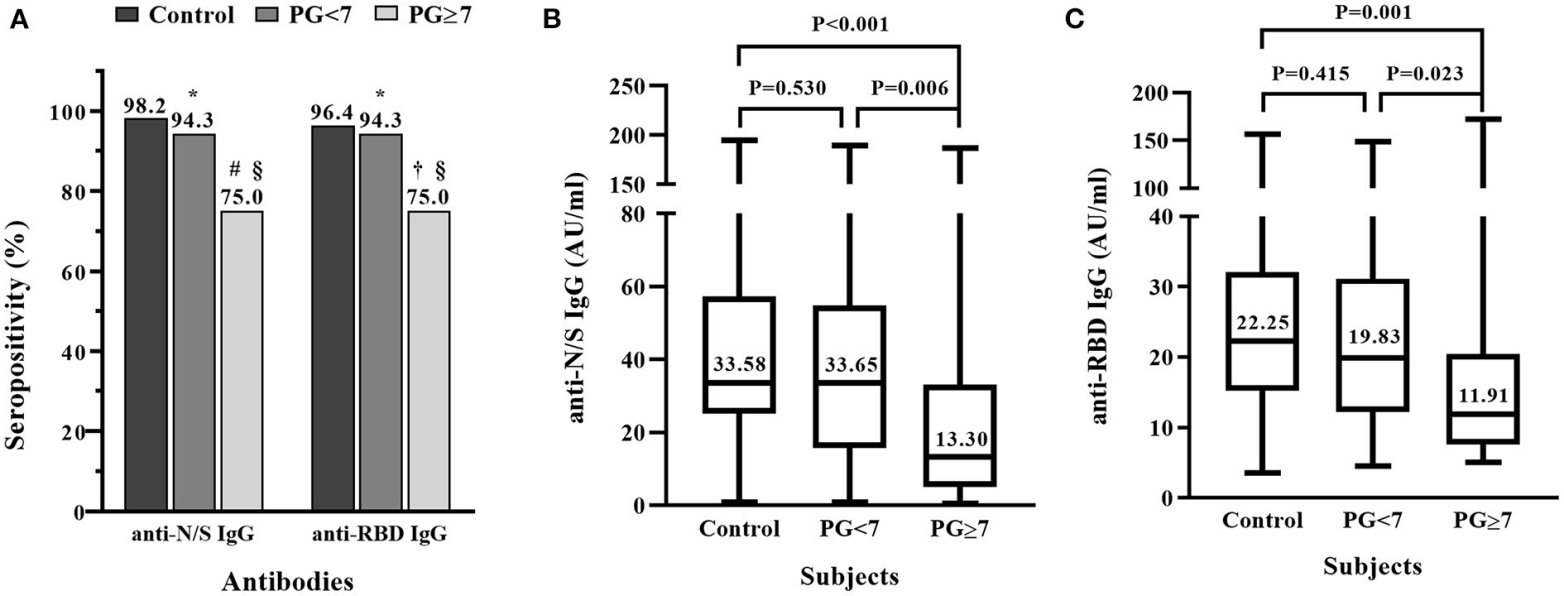
Comparison of IgG antibody response to inactivated COVID-19 vaccine in patients with diabetic mellitus (DM) who had controlled or uncontrolled fasting blood glucose and non-DM subjects. Thirty-five DM patients who had plasma glucose (PG) <7 mMol/L, 28 DM patients who had PG ≥7 mMol/L, and 56 non-DM subjects were each vaccinated with two doses of inactivated COVID-19 vaccine. **(A)** Positive rates of IgG antibody against the combination of nucleocapsid (N) and spike (S) proteins (anti-N/S IgG), and against receptor binding domain (RBD) (anti-RBD IgG). *P > 0.05, compared to control; ^#^*P* = 0.003, compared to control;^†^*P* = 0.009, compared to control; ^§^*P* = 0.030, comparison of diabetic patients with PG <7 and ≥7 mMol/L. **(B)** Comparison of titers of anti-N/S IgG between controls and diabetic patients with PG <7 and ≥7 mMol/L, respectively. **(C)** Comparison of titers of anti-RBD IgG between controls and diabetic patients with PG <7 and ≥7 mMol/L, respectively.

To further clarify whether the antibody response to COVID-19 vaccine is influenced by the uncontrolled glycemia, we compared the positive rates and the levels of anti-N/S IgG and anti-RBD IgG between T2DM patients who had controlled glycemia and those who had uncontrolled glycemia. Figure 2A shows that the positive rates of anti-N/S IgG and anti-RBD IgG in the T2DM patients with fasting blood glucose ≥7 mMol/L were both 75.0% (95% CI 55.1, 89.3%), significantly lower than the rates (94.3% [95% CI 80.8, 99.3%]) in the patients with fasting blood glucose <7 mMol/L (*P* = 0.030), and lower than those (anti-N/S IgG 98.2% and anti-RBD IgG 96.4%) in the non-T2DM individuals (*P* = 0.003 and 0.009, respectively). However, compared to non-diabetic controls, T2DM patients with fasting blood glucose <7 mMol/L had similar positive rate for anti-N/S IgG (94.3 vs. 98.2%, *P* = 0.676) and for anti-RBD IgG (94.3.0 vs. 96.4%, *P* = 1.000) (Figure 2A).

The comparison of antibody titers between the T2DM patients with controlled and uncontrolled glycemia and the non-T2DM individuals showed that the median levels of anti-N/S IgG and anti-RBD IgG in the patients with fasting blood glucose ≥7 mMol/L were much lower than those in the patients with fasting blood glucose <7 mMol/L, respectively (anti-N/S IgG: 13.30 vs. 33.65 AU/ml, *P* = 0.006; anti-RBD IgG: 11.91 vs. 19.83 AU/ml. *P* = 0.023), and significantly lower than those in the non-T2DM individuals (anti-N/S IgG: 13.30 vs. 33.58 AU/ml, *P* < 0.001; anti-RBD: 11.91 vs. 22.25 AU/ml. *P* = 0.001) (Figures 2B,C). However, the titers of anti-N/S IgG and anti-RBD IgG between the patients with fasting blood glucose <7 and non-T2DM subjects were comparable, respectively (anti-N/S IgG: 33.65 vs. 33.58 AU/ml, *P* = 0.530; anti-RBD IgG: 19.83 vs. 22.25 AU/ml, *P* = 0.415) (Figures 2B,C).

## Discussion

In the present study, we revealed that antibody response to COVID-19 vaccine composed of inactivated SARS-CoV-2 in T2DM patients was lower than that in non-T2DM subjects, and the antibody response in T2DM patients with uncontrolled glycemia was lower than that in T2DM patients with controlled glycemia. The data indicate that diabetic patients have reduced antibody response to inactivated COVID-19 vaccine, particularly in the patients with uncontrolled glycemia.

The participants included in this study were vaccinated with COVID-19 vaccine composed of inactivated SARS-CoV-2. Thus, the vaccinees were able to produce antibodies to all viral proteins of SARS-CoV-2. We used two types of assays to measure the antibody responses. One assay contains a combination of the N and S proteins of SARS-CoV-2, which can detect antibodies directed against both the N and S proteins. And the other assay contains the RBD domain only, which can detect antibodies specifically directed against RBD. Anti-RBD IgG antibodies are proved to be neutralizing against SARS-CoV-2 (39, 40). In the present study, 98.2% (55/56) and 96.4% (54/56) of the non-DM subjects showed anti-N/S IgG positive and anti-RBD positive, respectively after a full vaccination with two doses at an interval of 2–4 weeks (Table 1 and Figure 2), which is in agreement with the results in the clinical trials (33, 34). Thus, our data in the present study added more evidence that the inactivated COVID-19 vaccine efficiently elicited the non-neutralizing (anti-N) as well as neutralizing (anti-RBD) antibodies.

Diabetic patients are usually considered to be to some contents immunocompromised in both innate and adaptive immune responses. One of the common complications among diabetic patients is various infections (41). Clinical observations showed that COVID-19 patients who had underlying diabetes have an increased risk of severe disease and mortality (42, 43). This may be explained by the impaired antibody responses to the natural SARS-CoV-2 infection in diabetic patients (44), although others reported that diabetic patients with COVID-19 had same antibody responses as non-diabetic COVID-19 patients (45). In diabetic patients who were vaccinated with mRNA or viral vector-based COVID-19 vaccine, the antibody titers are relatively lower than that in non-diabetic subjects (26, 27). In our present study, we also observed that the antibody response to inactivated COVID-19 vaccine in diabetic patients was lower than that in subjects who had no diabetes. The impaired antibody response to inactivated COVID-19 vaccine was mainly seen in diabetic patients who had uncontrolled glycemia, whereas diabetic patients who had controlled glycemia showed similar antibody response as the non-diabetic subjects did (Figure 2). Our finding is in agreement with what reported by Marfella et al. that diabetic patients with poor glycemic control showed a weak immunity to mRNA vaccines (mRNA-BNT162b2 and mRNA-1273 vaccine) or a viral vector-based vaccine (ChAdOx1-S) (27). This suggests that uncontrolled glycemia may inhibit the immune response to COVID-19 vaccines. Indeed, compared to diabetic patients with controlled glycemia, those with poor glycemic control are at the increased risk of various infections (46).

The reduced antibody response (the seroconversion rate and antibody titers) to COVID-19 vaccine in diabetic patients observed in this study suggests that the protective efficacy and duration of protection against COVID-19 may be relatively lower, particularly in the patients with uncontrolled glycemia. Therefore, the issue of whether diabetic subjects with uncontrolled glycemia require more doses of COVID-19 vaccine, or a relatively shorter interval to receive booster immunization, to obtain the optimized protective efficacy merits further study. Alternatively, to have the full efficacy of the vaccine, diabetic patients with uncontrolled glycemia may delay the vaccination until their glycemia is controlled. Nevertheless, breakthrough infection occurred in diabetic patients who had already received COVID-19 vaccination is prone to have more severe COVID-19 than non-diabetic patients (47). Thus, other preventive measures, such as social distance and face masking, are still critical in diabetic subjects, even after COVID-19 vaccination.

There are several limitations in our study. First, it was single center study and the sample size was small. Second, the participants in this study received inactivated COVID-19 vaccines prepared by two manufacturers and we did not compare the antibody responses between these two inactivated vaccines. Third, although anti-RBD is considered to be neutralizing antibodies, we did not directly measure the neutralizing antibody response. Fourth, because of the limited number of patients with type 1 DM in the study period, we did not evaluate the antibody response to inactivated COVID-19 vaccines in such patients. Whether they have impaired immune response to COVID-19 vaccines requires further investigation.

In conclusion, after vaccinated with two doses of inactivated COVID-19 vaccines, T2DM patients with uncontrolled glycemia developed significantly lower anti-RBD IgG antibody levels than non-T2DM subjects and T2DM patients with controlled glycemia. Our results indicate that the vaccination schedule against COVID-19 requires further investigation to optimize the protective efficacy of COVID-19 vaccines.

## Data availability statement

The raw data supporting the conclusions of this article will be made available by the authors, without undue reservation.

## Ethics statement

The studies involving human participants were reviewed and approved by Institutional Review Board (IRB) of Nanjing Drum Tower Hospital (No. 2021-606-02). The patients/participants provided their written informed consent to participate in this study.

## Author contributions

YC, PS, ZH, and Y-HZ: conceptualized and designed the study. PS, YT, and WZ: performed the laboratory work. YC, YB, and ZH: recruited the study subjects. BX: participated in the design and performed the statistical analysis. YC and PS: drafted the manuscript. ZH and Y-HZ: critically revised the manuscript. All authors contributed to the interpretation of the results and agreed to the submitted version of the manuscript.
